# Associations Between Greek Affiliation, Parental Permissiveness Toward Heavy Episodic Drinking, and Alcohol Use Among First-Year College Students

**DOI:** 10.3390/bs15111488

**Published:** 2025-10-31

**Authors:** Kristi M. Morrison, Jennifer C. Duckworth, Matthew F. Bumpus, Martie L. Skinner, Brittany R. Cooper, Laura G. Hill, Kevin P. Haggerty

**Affiliations:** 1 Department of Human Development, Washington State University, Pullman, WA 99164, USA; jennifer.duckworth@wsu.edu (J.C.D.); brittany.cooper@wsu.edu (B.R.C.); laurahill@wsu.edu (L.G.H.); 2 Innovia Foundation, Spokane, WA 99201, USA; mbumpus@innovia.org; 3 Social Development Research Group, School of Social Work, University of Washington, Seattle, WA 98105, USAhaggerty@uw.edu (K.P.H.)

**Keywords:** parental permissiveness, fraternity and sorority, parent influence, college drinking, Greek affiliation

## Abstract

Parental permissiveness toward alcohol use is associated with increased drinking among college students. In the U.S., Greek-affiliated students drink more and experience more negative consequences than other students. This study explored associations among student Greek affiliation, parental permissiveness toward heavy episodic drinking (HED), and alcohol use outcomes among first-year college students. Parent-student dyads (*n* = 294) completed surveys during high school and the first semester of college at a large public university in the U.S. Paired- and independent-samples *t*-tests and regression analyses were conducted. Parental permissiveness toward HED was higher among Greek-affiliated students than non-Greek-affiliated students, from parent and student perspectives, before and during college. In regression analyses, student Greek affiliation and perceived parental permissiveness were associated with greater alcohol use and HED. Greek status moderated associations between perceived parental permissiveness of HED and alcohol use (but not HED) such that the relationship was less pronounced for Greek-affiliated students compared to non-Greek-affiliated students. Our results suggest that interventions that aim to reduce perceived parental permissiveness toward HED, such as parent-based normative feedback interventions, may be an effective strategy to reduce drinking among first-year Greek-affiliated students.

## 1. Introduction

High-risk alcohol use among college students is a significant public health concern. In the United States, about 52.1% of full-time college students report drinking in the past 30 days and about one quarter report consuming 5 or more drinks in a row in the past two weeks (Patrick et al., 2025). College students who engage in heavy episodic drinking (HED; 4+/5+ drinks in one occasion for females/males) are more likely to experience a number of alcohol-related consequences, including depression, sexual assault, injury, reduced academic performance, alcohol use disorder, and alcohol overdose (Patrick et al., 2020; Piazza-Gardner et al., 2016; Prince et al., 2019; A. White & Hingson, 2013). Furthermore, approximately 1500 college students die each year in the United States due to alcohol use (Hingson et al., 2017).

The transition to college is a developmental period marked by significant change for late adolescents such as moving away from home, changes in support networks, and new academic demands (Arnett, 2000; Schulenberg & Maggs, 2002). Incoming first-year students experience greatly reduced parental supervision while being introduced to social contexts characterized by increased access to alcohol (Borsari et al., 2007; Sher & Rutledge, 2007; H. R. White et al., 2005). When first-year students drink, they tend to engage in HED, and the risk of negative consequences escalates dramatically during the first semester of college (Riordan & Carey, 2019). First-year college students are also more likely to engage in high-risk sexual behavior, get in fights, visit the emergency room, and die as a result of HED than older students (Bailey et al., 2011; Borsari et al., 2007; Harford et al., 2003; Riordan & Carey, 2019).

Another important context for college drinking is membership in fraternities or sororities, which are common among U.S. college campuses. Fraternities (primarily for men) and sororities (primarily for women), also referred to as Greek organizations, are student-led social groups that provide opportunities for academic engagement, social events, community service, leadership development, shared traditions, and in some cases, shared housing. Although Greek membership among college students has been linked with many positive outcomes (e.g., increased sense of belonging, campus engagement, and academic performance; DeBard & Sacks, 2011; Hayek et al., 2002; Routon & Walker, 2014; Turton et al., 2018), Greek membership is also consistently identified as a risk factor for hazardous alcohol use and related negative consequences (Borsari et al., 2009; Ranker & Lipson, 2022). Indeed, relative to non-Greek members, students who enter fraternities and sororities tend to drink more often and engage in higher rates of HED than other students (Cashin et al., 1998; Caudill et al., 2006; Chauvin, 2012; McCabe et al., 2005; Ragsdale et al., 2012; Ranker & Lipson, 2022; Scott-Sheldon et al., 2008; Wechsler et al., 2009). They also experience more negative alcohol-related consequences, including injury, driving under the influence, sexual assault, police involvement, alcohol use disorder, and even death (Cashin et al., 1998; Chauvin, 2012; Grekin & Sher, 2006; McCabe et al., 2018; Ragsdale et al., 2012; Thompson & Huynh, 2017). Among Greek members, fraternity members tend to drink more frequently, consume greater quantities of alcohol, and experience more negative alcohol consequences than sorority members (Brown-Rice et al., 2015; Larimer et al., 2000). Furthermore, fraternity involvement during college is associated with increased odds of alcohol use disorder in early midlife (McCabe et al., 2018).

Previous research suggests that heavy drinking in the Greek system is driven primarily by two mechanisms: selection and socialization effects (Borsari et al., 2009; McCabe et al., 2005; Park et al., 2009). Selection refers to the process by which individuals who have certain characteristics will be more likely to self-select into environments that support those characteristics. For example, students who join fraternities and sororities tend to engage in greater alcohol use and experience more alcohol problems prior to college (Capone et al., 2007; Park et al., 2009). Socialization, in contrast, refers to the process by which being in a social environment where alcohol use and HED are prevalent and accepted can influence individuals to adopt similar behaviors and attitudes. For example, several studies have identified that Greek affiliation is associated with increased alcohol use and problems during college (Capone et al., 2007; McCabe et al., 2005). Furthermore, research suggests that selection and socialization can occur simultaneously, influencing each other over time (Park et al., 2009).

Given the increased risk of HED and negative consequences for first-year students and for students in fraternities or sororities, it is important to explore how risk and protective factors may exacerbate or buffer hazardous drinking for these groups. Parents continue to play an important role in their lives during college, especially during the transition to college (Dorrance Hall et al., 2016; Kenny, 1987; Lowe & Dotterer, 2017; Wintre & Yaffe, 2000). Several parenting behaviors and characteristics have been identified as protective or risk factors for college student alcohol use. For example, frequent parent-student communication is associated with reduced alcohol use (Hamilton et al., 2021; Small et al., 2011; Trager et al., 2023a), whereas perceived permissive parental attitudes toward alcohol use have been linked with increased student alcohol use (e.g., Boyle & Boekeloo, 2006; Calhoun et al., 2018; Walls et al., 2009).

Social norms theory suggests that an individual’s behavior is influenced by their perception of how other members in their social groups, including their parents, think or behave (Berkowitz, 2004; Neighbors et al., 2007). College students tend to overestimate how approving others (e.g., parents and peers) are of alcohol use, and these overestimations are related to increased student drinking (LaBrie et al., 2010; Larimer et al., 2004; Neighbors et al., 2007; Pedersen et al., 2017). A large body of research links student alcohol use and perceived parental permissiveness toward drinking. Throughout the college years, students whose parents are more approving of their alcohol use tend to drink more and experience more negative alcohol-related consequences than students whose parents are less approving (e.g., C. Abar et al., 2009; Calhoun et al., 2018; Mallett et al., 2019; Varvil-Weld et al., 2014). Differences in perception of permissive parental attitudes by student gender have been documented such that compared to female students, male students perceive their parents to be more permissive toward their drinking (Calhoun et al., 2018). Moreover, a growing body of research indicates that parent-focused interventions during the college years can be effective at reducing risk behaviors including substance use (Hill et al., 2023; LaBrie et al., 2016; Stormshak et al., 2019; Turrisi et al., 2001). Better understanding how perceived parental permissiveness toward HED among fraternity and sorority members relates to alcohol use during the transition to college may be an important step for prevention and intervention efforts aimed at decreasing hazardous use among fraternity/sorority members.

Despite the continued importance of parents during the college years and the potential hazards of fraternity/sorority membership in terms of alcohol use, little research has focused on parents of fraternity/sorority members (Larimer et al., 2000; Turner et al., 2000), and, to our knowledge, no studies have examined parental permissiveness toward alcohol or its association with alcohol use among Greek-affiliated students. It remains unclear whether students who join fraternities and sororities differ from their peers prior to joining (selection effects) or whether involvement in these organizations shapes subsequent drinking behaviors (socialization effects). For instance, students who join fraternities and sororities may have grown up in families with more permissive attitudes toward alcohol use. Although selection effects are typically used to describe student attitudes and behaviors, parental permissiveness toward drinking may function in a similar way such that students whose parents are more approving of drinking may be more likely to self-select into environments that reinforce similar drinking attitudes, including Greek organizations.

Previous research suggests that parent-based normative feedback interventions that aim to correct misperceptions about parental permissiveness toward alcohol use can be effective at reducing alcohol use and HED among college students by reducing students’ perception of parental permissiveness of alcohol use (LaBrie et al., 2016; LaBrie et al., 2022). If perceived parental permissiveness toward alcohol use is a risk factor for alcohol use and HED for fraternity and sorority members as it is for college students more broadly, parent-based normative feedback interventions may be an effective strategy to reduce drinking among this high-risk group.

The present study has two primary aims. The first aim was to examine differences in parental permissiveness of HED by student Greek affiliation, from the perspective of both the student and parent. Findings from this aim could inform the adaptation of parent-based interventions for Greek-affiliated students. Given the lack of research on permissive parental attitudes toward alcohol use among Greek-affiliated students, we considered Aim 1 to be exploratory. The second aim was to examine associations between Greek affiliation, perceived parental permissiveness toward HED, and frequency of alcohol use and HED (Aim 2) among first-year college students. As a part of this aim, we examined whether Greek affiliation moderated associations between perceived parental permissiveness and alcohol use outcomes. Based on prior research, we hypothesized that both Greek affiliation and perceived parental permissiveness toward HED would be positively related to alcohol use outcomes. Tests of moderation were exploratory.

## 2. Materials and Methods

### 2.1. Participants and Procedures

The analytic sample comes from a longitudinal study testing the effectiveness of a self-directed handbook intervention for parents of college students aimed at promoting positive academic outcomes and preventing risk behaviors by increasing family protective factors. Students who met eligibility criteria (attending college for the first time in the fall semester, under age 21, English-speaking, and currently residing in the United States) were randomly selected from the admitted first-year population at a large public university in 2017 and 2018, in two consecutive cohorts. Students and one parent or caregiver (hereafter referred to as “parent”) were randomly assigned to one of three conditions: control, handbook, or handbook plus booster messages. Families were recruited directly into their assigned condition. Both student and parent provided consent and completed a baseline survey to be included in the study (see Cooper et al., 2020 for more details). The final sample of students was representative of the university’s first-year student population in terms of gender, race/ethnicity, and first-generation college status.

Students and parents completed surveys during the spring of the student’s senior year of high school (baseline) and again four months later, during the fall of their first semester of college (Time 2; approximately 1–2 months after the semester started). Participants received a $20 gift card upon survey completion. Because the intervention is associated with reduced alcohol use among first-year college students, which is one of our outcome variables, the analytic sample for the current study is based on data collected from the normative sample of control participants only (*n* = 309). Of these, 294 students (95.2%) completed both baseline and Time 2 surveys. All study procedures were approved by the university’s Internal Review Board.

### 2.2. Measures

#### 2.2.1. Parental Permissiveness Toward HED

At baseline (i.e., spring of the student’s senior year of high school) and again at Time 2 (i.e., fall of the student’s first semester of college), parents were asked how wrong they felt it would be for their student to engage in HED (4+/5+ drinks in one occasion for females/males) once or twice a week. Response options were: 1 (very wrong), 2 (wrong), 3 (a little bit wrong), and 4 (not at all wrong). This measure was adapted from the Communities That Care Youth Survey, which has been studied extensively, with high levels of reliability and validity (Arthur et al., 2002).

#### 2.2.2. Perceived Parental Permissiveness Toward HED

At baseline and Time 2, students were asked to assess how wrong they thought their parents felt it would be for them to engage in HED once or twice a week. Again, response options ranged from 1 (very wrong) to 4 (not at all wrong; Arthur et al., 2002).

#### 2.2.3. Student Greek Affiliation

At Time 2, students were asked “are you a member of a fraternity or sorority” (0 = no, 1 = yes), approximately 1–2 months after fraternity and sorority recruitment.

#### 2.2.4. Alcohol Use and HED

At baseline and Time 2, students were asked on how many occasions they drank alcohol in the past 30 days and how often they had four or more (females) or five or more (males) alcoholic drinks in a row in the past two weeks. For both questions, response options were rated on a 7-point ordinal scale including 0 (0 occasions), 1 (1 to 2 occasions), 2 (3 to 5 occasions), 3 (6 to 10 occasions), 4 (11 to 19 occasions), 5 (20 to 39 occasions), and 6 (40 or more occasions).

#### 2.2.5. Demographics

Students provided demographic information on their university admissions application. Specifically, students reported their age, gender (0 = male, 1 = female), race and ethnicity, and parents’ level of education. Based on parents’ level of education, first-generation college status was coded as 0 (at least one parent has earned a bachelor’s degree) or 1 (neither parent has earned a bachelor’s degree).

### 2.3. Analytic Strategy

All analyses were conducted using SAS 9.4 software (SAS Institute, 2014). First, we examined descriptive statistics as part of preliminary analyses. As part of preliminary analyses, we also examined the change in parental permissiveness toward HED, perceived parental permissiveness toward HED, and frequency of alcohol use and HED from baseline to Time 2 using paired-samples *t*-tests. We also used bivariate correlations to assess the relationship between parent and student report of parental permissiveness of HED.

#### 2.3.1. Aim 1: Differences in Parental Permissiveness Toward HED by Greek Affiliation

To address aim 1, we conducted independent-samples *t*-tests to assess differences in parental permissiveness toward HED by student Greek affiliation based on parent and student report at baseline and Time 2.

#### 2.3.2. Aim 2: Associations Among Perceived Parental Permissiveness Toward HED, Greek Affiliation, and Frequency of Alcohol Use Outcomes

Due to a skip-pattern programming error, 44 respondents who reported past-30-day alcohol use were not administered the HED item at baseline and were excluded from HED analyses. Missingness was unrelated to most demographic variables (gender, age, first-generation college status) and parental permissiveness of HED but was somewhat less common among Black students and was associated with greater alcohol use frequency.

Because past-30-day alcohol use and past-two-week HED were both positively skewed, we used negative binomial regression models to examine associations between Greek affiliation and perceived parental permissiveness of HED with frequency of alcohol use or HED. Models controlled for baseline alcohol use or HED, gender, race/ethnicity, and first-generation college status. High school (baseline) alcohol use or HED was included as a covariate because it was strongly associated with college (Time 2) alcohol use and HED. Gender, race/ethnicity, and first-generation college status were included as covariates because previous research indicates that student Greek affiliation, perceived parental permissiveness of alcohol, and alcohol use vary by these demographic variables (Calhoun et al., 2018; Caudill et al., 2006; DiGuiseppi et al., 2020; Patrick et al., 2025). Due to small sample sizes, American Indian/Alaska Native and Native Hawaiian/Other Pacific Islander participants were combined into an “other” category.

In models examining alcohol use outcomes, we examined only students’ perception of parental permissiveness toward HED and not parents’ reports of parental permissiveness toward HED, as previous research suggests that students’ reports of parenting behaviors are more consistently associated with alcohol use outcomes (e.g., Trager et al., 2023b; Varvil-Weld et al., 2013). For example, Varvil-Weld et al. (2013) found that students’ reports of permissiveness predicted student alcohol use and consequences more reliably than parents’ reports. Finally, we explored Greek affiliation as a potential moderator of the relationship between perceived parental permissiveness toward HED and alcohol use or HED by including the interaction between Greek affiliation and perceived parental permissiveness toward HED in models described above.

## 3. Results

### 3.1. Descriptive Results

Descriptive results for the full sample and by Greek affiliation are displayed in Table 1. At baseline, the mean age of students was 18.16 years (SD = 0.35) and 60.2% identified as female. About one third (*n* = 95) of students reported joining a fraternity or sorority in the fall of their first year of college (i.e., Time 2). Among parents, 79.93% of participants were mothers, 19.73% were fathers, and <1% were other caregivers. On average, parents reported that it was between ‘very wrong’ and ‘wrong’ for their students to engage in HED at both baseline (*M* = 1.33, *SD* = 0.58) and Time 2 (*M* = 1.44, *SD* = 0.73). At baseline, students perceived that their parents felt it was between ‘very wrong’ and ‘wrong’ for students to engage in HED (*M* = 1.89, *SD* = 0.91), and at Time 2, between ‘wrong’ and ‘a little bit wrong’ for students to engage in HED (*M* = 2.11, *SD* = 0.97). Parental permissiveness toward HED increased significantly from baseline to Time 2 from parents’ perspective (*t* = 2.68, *p* = 0.008) and students’ perspective (*t* = 3.61, *p* < 0.001). Correlations between parental permissiveness toward HED and student perception of parental permissiveness toward HED were positive and weak at baseline (*r* = 0.21, *p <* 0.001) and Time 2 (*r* = 0.27, *p <* 0.001).

### 3.2. Aim 1: Differences in Perceived Parental Permissiveness Toward HED and Alcohol Use by Greek Affiliation

We observed significant differences by Greek affiliation in parental permissiveness toward HED and perceived parental permissiveness toward HED (Table 1). Specifically, parents of Greek-affiliated students reported being more permissive toward their student’s HED than parents of non-Greek-affiliated students both at baseline (*t* = −3.58, *p* < 0.001) and Time 2 (*t* = −5.11, *p* < 0.001). Similarly, Greek-affiliated students perceived their parents to be more permissive toward HED than did their non-Greek-affiliated peers at baseline (*t* = −5.81, *p* < 0.001) and Time 2 (*t* = −7.2, *p* < 0.001).

### 3.3. Aim 2: Associations Among Greek Affiliation, Perceived Parental Permissiveness Toward HED, and Frequency of Alcohol Use and HED

Rate ratios for the associations between Greek affiliation, perceived parental permissiveness toward HED, and alcohol use and HED are presented in Table 2. Results from negative binomial regression models indicate that student Greek affiliation was positively associated with past-30-day alcohol use frequency [RR (95% CI) = 1.94 (1.58–2.39)] and past-two-week HED frequency [RR (95% CI) = 2.32 (1.69–3.2)] at Time 2, controlling for baseline alcohol or HED, perceived parental permissiveness, and demographic variables. Perceived parental permissiveness toward HED at Time 2 was positively associated with alcohol use [RR (95% CI) = 1.18 (1.07–1.31)] and HED [RR (95% CI) = 1.49 (1.26–1.75)] at Time 2, controlling for covariates. In subsequent analyses, the interaction between perceived parental permissiveness toward HED at Time 2 and student Greek affiliation was significantly associated with alcohol use (but not HED) at Time 2 (*β* = −0.25, *p* = 0.013), indicating that the relationship between perceived parental permissiveness of HED and alcohol use frequency was less pronounced for Greek-affiliated students.

## 4. Discussion

Due to the convergence of multiple risk factors, the transition to college is often associated with sharp increases in alcohol use, including HED, and negative alcohol-related consequences (Borsari et al., 2007; Sher & Rutledge, 2007). This pattern is exacerbated for those who join a fraternity or sorority (Ragsdale et al., 2012). Given the scarcity of research on parents of Greek-affiliated students, we first examined differences by Greek affiliation in parental permissiveness toward HED and alcohol use during the first semester of college. Next, we explored the relationship among Greek affiliation, perceived parental permissiveness toward HED, and alcohol use outcomes.

Consistent with previous research regarding alcohol use, we found that parents, on average, became more permissive toward their students’ HED across the transition to college (Calhoun et al., 2018). The pattern of increased parental permissiveness held true from the perspectives of both parents and students. Although some parents may adjust their expectations as their student enters environments where alcohol use is more common, it is important to note that all participants were under the legal drinking age. Moreover, research suggests that compared to permissive or harm reduction messages, zero-tolerance alcohol messages from parents are associated with lower alcohol use and related consequences among college students (C. C. Abar et al., 2012; LaBrie et al., 2015; Miller-Day, 2008; Napper, 2019). Thus, students may benefit from parents more clearly communicating their disapproval of HED and their expectations for their students’ alcohol use in college (C. C. Abar et al., 2011; Miller-Day, 2008; Reimuller et al., 2011).

We also found low concordance between parental permissiveness toward HED and student perception of parental permissiveness toward HED. These findings suggest a disconnect between what students perceive parents think and what parents actually think. One potential explanation is that parents are not explicitly communicating their attitudes toward and expectations around alcohol use to their students. Additionally, a parent’s behavior, such as parental alcohol use, may inform student perceptions of parental permissiveness more than explicit conversations about alcohol (Kam et al., 2017). It is also possible, however, that the low concordance reflects a measurement limitation, as students reported on their parents’ permissiveness, not the parent who completed the survey. Taken together, these results suggest a need for clear communication from parents to their offspring regarding their disapproval of HED and their expectations for their students’ own alcohol use (C. C. Abar et al., 2011; Miller-Day, 2008; Reimuller et al., 2011).

When exploring the role of students’ Greek affiliation in parent-reported and student-perceived parental permissiveness toward HED, we found that Greek-affiliated students and their parents reported greater permissiveness toward HED in high school and college relative to non-Greek-affiliated students and their parents. This finding suggests that students who join fraternities and sororities come from families with more permissive attitudes toward drinking. It is possible that these permissive attitudes may impact a student’s decision to associate with environments that are more permissive towards alcohol such as in the Greek system (i.e., selection effects). Coupled with prior findings that parental permissiveness changes across the college transition, permissiveness may serve both as a pre-existing family factor linked to selection into more permissive social environments and as a dynamic construct that shifts during the transition to college. Recognizing this dual role highlights that parental attitudes toward alcohol are not fixed but may evolve in response to developmental and contextual changes.

We also examined the relationship among Greek affiliation, perceived parental permissiveness toward HED, and alcohol use during the first semester of college. We focused on student perception of parental permissiveness, as students’ reports of parenting attitudes are more reliably associated with alcohol use (Trager et al., 2023b; Varvil-Weld et al., 2013). Consistent with previous research (C. Abar et al., 2009; Calhoun et al., 2018; Cashin et al., 1998; Chauvin, 2012; Mallett et al., 2019; Ragsdale et al., 2012; Scott-Sheldon et al., 2008; Varvil-Weld et al., 2013; Walls et al., 2009; Wechsler et al., 2009), we found that both Greek affiliation and perceived parental permissiveness toward HED were positively associated with alcohol use outcomes.

Even after controlling for Greek affiliation, perceived parental permissiveness remained strongly associated with student alcohol use and HED, suggesting that parental permissiveness is a risk factor for both Greek and non-Greek-affiliated students. This highlights parental permissiveness as a potential target for interventions designed to reduce alcohol use among college populations more broadly. Prior intervention research suggests that targeting parental norms can influence college student alcohol use outcomes. For example, Labrie and colleagues (LaBrie et al., 2016; LaBrie et al., 2014; LaBrie et al., 2022) developed a parent-based normative feedback intervention to correct parent misperceptions of how approving other parents are of student alcohol use (which parents tend to overestimate), how much other parents communicate with their child about alcohol (which parents tend to underestimate), and how much their student would drink in college (which parents tend to underestimate). Results from these studies suggest that students whose parents participated in the intervention engaged in less alcohol use and HED in college compared to students in the control condition, and this was mediated by students’ lowered perception of parent permissiveness toward alcohol use. Consistent with Borsari et al. (2009)’s recommendations, parent-based interventions that aim to decrease parental approval of heavy drinking (e.g., LaBrie et al., 2016; LaBrie et al., 2022) may be a promising strategy to reduce drinking among Greek-affiliated students, a high-risk group. Further work is needed to determine whether changes in perceived permissiveness translate into reduced alcohol use in this population.

This study found that the relationship between perceived parental permissiveness toward HED and frequency of alcohol use, but not HED, was less pronounced for Greek-affiliated students relative to non-Greek-affiliated students. The moderation effect may have emerged for alcohol use but not HED, because HED was less commonly endorsed and had a smaller sample size, reducing power to detect moderation effects. Given the social context of fraternities and sororities, it is possible that peers exert a greater influence on student alcohol use than parents do. For example, previous research suggests that perceived peer drinking norms are important predictors of alcohol use among fraternity and sorority members (Capone et al., 2007). Future research should explore the relative influence of parent and peer norms on alcohol use among Greek-affiliated students (Wood et al., 2004). Interventions targeting both parent and peer alcohol norms among Greek-affiliated students may be especially promising for reducing use among this high-risk population (Borsari et al., 2009).

This study has several strengths, including a representative sample of a large public university’s first-year student population and low attrition rates. Additionally, we collected data from both parents and students at two different timepoints, allowing us to assess both parents’ report and students’ perceptions of parental HED permissiveness in the spring of the student’s senior year of high school, prior to the student’s departure for college, as well as during the first semester of college.

Despite these strengths, the study should be considered in light of limitations. Parental permissiveness toward HED and student perception of parental permissiveness toward HED were each assessed using a single indicator, which, while not uncommon in the literature (C. Abar et al., 2009; Calhoun et al., 2018), offers only a limited assessment and is more susceptible to measurement error. Due to the skip-pattern error, students who engaged in HED may be underrepresented in analyses examining HED. This limitation could attenuate observed associations between parental permissiveness, Greek affiliation, and HED frequency, including moderation effects. Students reported on their parents’ permissiveness in general, not specifically the attitudes of the parent who completed the parent survey, which may have led to lower parent-student consistency. Generalizability may be limited as participants were recruited from a single residential university, families willing to participate in family-based research may not be representative of all families, and results from these cohorts may not reflect current students. We did not assess whether students’ parents were Greek members, which could influence permissiveness and students’ Greek affiliation. Finally, because the study is correlational, we cannot draw causal conclusions.

In sum, the early weeks of college are a particularly risky time for heavy drinking, especially for those who enter a fraternity or sorority. Not only do Greek-affiliated students engage in more alcohol use than other students, but Greek-affiliated students perceive that their parents are more permissive toward their alcohol use. Our results suggest that perceived parental permissiveness toward HED may be an effective risk factor for interventions to target in order to reduce high-risk drinking in first-year Greek-affiliated students. Future research should examine the efficacy of parent-based interventions for parents of Greek-affiliated students, such as normative feedback interventions.

## Figures and Tables

**Table 1:** Demographic Characteristics and Parental Permissiveness Toward HED by Full Sample, Greek-affiliated Students, and Non-Greek-affiliated Students.

	Full Sample (*n* = 294)	Greek-Affiliated Students (*n* = 95, 32.31%)	Non-Greek-Affiliated Students (*n* = 199, 67.69%)	Greek vs. Non-Greek-Affiliated
	% or M (SD)	% or M (SD)	% or M (SD)	Test Statistic
Female	60.20	57.89	61.31	*X*^2^ = 0.31
Race and ethnicity				*X*^2^ = 19.13 **
American Indian/Alaska Native	1.37	3.23	0.51	
Asian	4.81	1.08	6.57	
Black/African American	5.15	3.23	6.06	
Hispanic/Latino	19.24	11.83	22.73	
Native Hawaiian/Other Pacific Islander	0.34	0.00	0.51	
White	53.95	68.82	46.97	
Two or more races	15.12	11.83	16.67	
First-generation status	34.81	28.42	37.88	*X*^2^ = 2.53
Parental permissiveness toward HED at baseline	1.33 (0.59)	1.53 (0.71)	1.24 (0.49)	*t* = −3.58 ***
Parental permissiveness toward HED at Time 2	1.44 (0.73	1.78 (0.87)	1.28 (0.58)	*t* = −5.11 ***
Perceived parental permissiveness toward HED at baseline	1.89 (0.91)	2.32 (0.93)	1.69 (0.84)	*t* = −5.81 ***
Perceived parental permissiveness toward HED at Time 2	2.11 (0.97)	2.65 (0.90)	1.85 (0.89)	*t* = −7.20 ***

Note. Baseline = spring semester of senior year of high school; Time 2 = fall semester of first year of college; HED = Heavy episodic drinking. ** *p* < 0.01. *** *p* < 0.001.

**Table 2:** Rate Ratios (and 95% Confidence Intervals) for Associations Among Student Greek Affiliation, Parental Permissiveness Toward HED, and Frequency of Alcohol Use and HED.

	Frequency of Past-30-Day Alcohol Use at Time 2 (*n* = 293)		Frequency of Past-Two-Week HED at Time 2 (*n* = 249)	
	Rate Ratio	95% CI	Rate Ratio	95% CI
First-generation status	0.88	(0.72, 1.08)	0.89	(0.65, 1.21)
Hispanic/Latino	0.86	(0.65, 1.13)	1.11	(0.71, 1.74)
Black/African American	0.86	(0.53, 1.38)	0.82	(0.42, 1.62
Asian	1.44	(0.94, 2.21)	1.00	(0.46, 2.22)
Other race	0.73	(0.36, 1.48)	0.44	(0.06, 3.16)
Two or more races	1.09	(0.83, 1.44)	0.95	(0.61, 1.48)
Female	1.16	(0.96, 1.40)	1.05	(0.78, 1.41)
Baseline alcohol use	1.19 ***	(1.11, 1.28)	--	--
Baseline HED	--	--	1.43 ***	(1.21, 1.69)
Student Greek affiliation	1.94 ***	(1.58, 2.39)	2.32 ***	(1.69, 3.20)
Perceived parental permissiveness toward HED at Time 2	1.18 **	(1.07, 1.31)	1.49 ***	(1.26, 1.75)

Note. Baseline = spring semester of senior year of high school; Time 2 = fall semester of first year of college; HED = Heavy episodic drinking. ** *p* < 0.01. *** *p* < 0.001.

## Data Availability

The data presented in this study are available upon request from the corresponding author due to privacy restrictions.
